# G: Fracture energy, friction and dissipation in earthquakes

**DOI:** 10.1007/s10950-016-9560-1

**Published:** 2016-03-31

**Authors:** S. Nielsen, E. Spagnuolo, M. Violay, S. Smith, G. Di Toro, A. Bistacchi

**Affiliations:** 1 Istituto Nazionale di Geofisica e Vucanologia, Rome, Italy; 2 Durham University, Earth Sciences, Durham, UK; 3 EPFL, Lausanne, Switzerland; 4 Department of Geology, University of Otago, Otago, New Zealand; 5 School of Earth, Atmospheric and Environmental Sciences, Manchester University, Manchester, UK; 6 Dipartimento di Geoscienze Address, Università degli Studi di Padova Division, Padova, Italy; 7 Department of Earth and Environmental Sciences, Universitá degli Studi di Milano Bicocca, Milan, Italy

**Keywords:** Earthquake scaling, Fracture energy, Laboratory experiments, High velocity friction

## Abstract

Recent estimates of fracture energy *G* ^′^ in earthquakes show a power-law dependence with slip *u* which can be summarized as *G* ^′^ ∝ *u* ^*a*^ where *a* is a positive real slightly larger than one. For cracks with sliding friction, fracture energy can be equated to *G*_*f*_: the post-failure integral of the dynamic weakening curve. If the dominant dissipative process in earthquakes is friction, *G* ^′^ and *G*_*f*_ should be comparable and show a similar scaling with slip. We test this hypothesis by analyzing experiments performed on various cohesive and non-cohesive rock types, under wet and dry conditions, with imposed deformation typical of seismic slip (normal stress of tens of MPa, target slip velocity > 1 m/s and fast accelerations ≈ 6.5 m/s^2^). The resulting fracture energy *G*_*f*_ is similar to the seismological estimates, with *G*_*f*_ and *G* ^′^ being comparable over most of the slip range. However, *G*_*f*_ appears to saturate after several meters of slip, while in most of the reported earthquake sequences, *G* ^′^ appears to increase further and surpasses *G*_*f*_ at large magnitudes. We analyze several possible causes of such discrepancy, in particular, additional off-fault damage in large natural earthquakes.

## Introduction

Seismic rupture is controlled by an energy balance involving elastic work, dissipation by anelastic processes (friction, damage, and plastic strain) and wave radiation. Creation or reactivation of rupture within a solid requires an energy input *G* (J m^−2^) per unit surface, called material toughness or fracture energy (*G* may be envisioned, for example, as the energy spent in severing atomic bonds). Griffith (1921) originally proposed a rupture propagation criterion based on the balance, during rupture advancement, between the energy made available by release of elastic stress and the surface energy necessary to create fresh crack face. Irwin (1957) later proposed to add plastic strain energy around the crack tip in the balance. Since then, the energy balance concept was expanded and developed in different forms (Rice 1968) and applied to a wide variety of problems including engineering applications (Broberg 1999), rock mechanics (Atkinson and Meredith 1987), and earthquake physics (Ida 1972; Andrews 1976; Aki 1979; Wong 1982; Ohnaka et al. 1986; Andrews 2005; Lancieri et al. 2012; Malagnini et al. 1994).

During earthquakes, stress is rapidly released because of a drop in friction, causing high (≈1 m/s) slip velocity, high (several km/s) rupture velocity, and wave radiation. Friction drops with slip and slip velocity, a process known as weakening. The simplest and most widely adopted model for frictional weakening, introduced by Ida (1972), consists in a linear decrease of the friction coefficient with slip from peak *μ*_*p*_ to a dynamic, steady-state *μ*_*ss*_, over a slip distance *D*_*c*_. (The corresponding shear stress drops from *τ*_*p*_ = *μ*_*p*_ *σ*_*n*_ to *τ*_*ss*_ = *μ*_*ss*_ *σ*_*n*_, where *σ*_*n*_ is the fault-normal stress). The weakening distance *D*_*c*_ has been widely used in fault studies and often imposed as a constant in fault models (Andrews 1976, and references therein; Mai et al. 2006). However, several authors have argued that *D*_*c*_ should be treated as a variable (Cocco and Bizzarri 2002; Nielsen et al. 2010), since the effective weakening distance depends on slip history, loading conditions, and is likely to become dynamically determined during rupture itself. It is recognized (Palmer and Rice 1973) that frictional work (product of shear stress and slip) during the weakening process equates to a particular form of fracture energy, such that many earthquake studies have adopted the simple form

1 $$ G=\frac{1}{2}(\tau_{p}-\tau_{ss})\ D_{c}\ ,  $$

assuming Ida’s slip weakening law, whereas the slightly more general form

2 $$ G(u)={{\int}_{0}^{u}}\left( \tau(u^{\prime})-\tau(u)\right)du^{\prime}\ ,  $$

can take into account forms of non-linear friction decay and considers the possibility that final slip *u* < *D*_*c*_ and the shear stress remains higher than the lowest possible dynamic value (Abercrombie and Rice 2005).

Laboratory measures of *D*_*c*_ obtained under different conditions of sliding velocity and normal stress show very large variations. On the one hand, rate and state friction (RAS) parameters (Dieterich 1979; Ruina 1983) obtained by velocity-stepping at sub-seismic slip rates indicate an evolution distance 10^−6^ < *L* < 10^−4^ m, and a modest (a few percent or less) friction drop. Rupture modeling shows (Guatteri et al. 2001; Tinti et al. 2004) that, due to the complexity in the slip history, a fixed parameter *L* in the RAS law can result in different effective slip weakening distances *D*_*c*_. However, the difference between *D*_*c*_ and *L* is at most 50 % and both well below 1 mm. On the other hand, rotary friction experiments performed at seismic (≈1 m/s) slip rates yield weakening distances in the order of meters, substantial friction drops (between 50 and 95 %) and fracture energies arguably in the same order as the seismological estimates (see discussion in further sections).

Seismological estimates of *D*_*c*_ suffer from poor resolution and from a fundamental indetermination: rupture is mainly sensitive to *G* (Peyrat et al. 2004; Spudich and Guatteri 2000) which is in essence the product of stress drop (*τ*_*p*_−*τ*_*ss*_) and *D*_*c*_, as shown in Eq.1, so that both cannot be determined independently. Exceptional circumstances may allow an unbiased constraint of *D*_*c*_ in earthquakes, like the recording of radiation from a patch of fault where rupture is propagating at supershear velocity (i.e., exceeding shear wave velocity), as argued by Cruz-Atienza and Olsen (2010).

Ultimately, fault properties which control rupture propagation are better represented by fracture energy *G* rather than by individual aspects of the weakening process. In addition, *G* appears in the energy balance (see later discussion around Eq. 15) against radiated energy (which can be estimated from detected waves) and elastic energy release (which can be estimated from final fault slip). As a consequence, under a series of assumptions, it is possible to obtain estimates of *G* for earthquakes and its variation with seismic moment and slip, as attempted by several authors (Aki 1979; Abercrombie and Rice 2005; Tinti E. et al. 2005; Malagnini et al. 1994; Causse et al. 2014; Viesca and Garagash 2015).

The strain energy release rate $\mathcal {G}$ (J m^−2^) is defined as the energy (originating from elastic strain release) made available per unit of newly created fracture surface area. In dynamic rupture propagation, $\mathcal {G}$ depends on rupture velocity *V*_*r*_, and $\mathcal {G}(V_{r})$ can be defined analytically for simple cases of cracks or slip pulses propagating at constant velocity (Kostrov 1974; Nielsen and Madariaga 2003; Rice et al. 2005). The simplest example is for the anti-plane (Mode II) crack where $\mathcal {G=}\mathcal {G}_{0}\sqrt {1-(V_{r}/V_{S})^{2}}$; $\mathcal {G}_{0}$ is the elastic strain energy release for a quasi-static crack (in the limit of vanishing small rupture velocity *V*_*r*_) and *V*_*S*_ is the shear wave velocity. The square root term is the well-known Lorentz contraction encountered also in special relativity. Though no closed mathematical expression for $\mathcal {G}$ exists for general cases, $\mathcal {G}(V_{r})$ is generally a monotonously decreasing function of *V*_*r*_ for sub-Rayleigh rupture velocities (*V*_*r*_ < *V*_*Ral*_ ), but can be more complex at intersonic rupture velocities (*V*_*S*_ < *V*_*r*_ < *V*_*P*_) (Broberg 1999); $\mathcal {G}$ is maximized and equates to $\mathcal {G}_{0}$ at low rupture velocity. For the purpose of earthquake studies, the criterion for rupture advancement is $\mathcal {G}(V_{r})=G$, where *G* is the material toughness (or fracture energy) of the material. When a rupture stops (e.g., at the end of earthquake rupture propagation), the maximum available strain energy release does not suffice to compensate the dissipation through fracture energy *G*; as a consequence, the condition $G\ge \mathcal {G}_{0}$ is verified. For a shear crack of radius *a* with static stress drop Δ*σ* = *τ*_0_−*τ*_1_ (where *τ*_0_ is initial and *τ*_1_ final shear stress) and a shear stiffness *μ* ^′^, the static fracture energy release rate is

3 $$ \mathcal{G}_{0}=\frac{\pi\ {\Delta}\sigma^{2}a}{\mu^{\prime}}  $$

Relation 3 was used by Aki (1979) to derive for the first time a lower bound estimate of material toughness *G* for an earthquake fault barrier (the radius *a* was inferred from the perimeter of aftershock activity around an asperity). On the other hand, the well-known scaling of slip with fault size and stress drop is described by

4 $$ u=C_{1}\ \frac{\Delta\sigma}{\mu^{\prime}}\ a $$

(*C*_1_ is a dimensionless constant of order unity, depending on the shape of the ruptured area). Replacing *a* from Eq. 4 into 3, and using *C* = *π*/*C*_1_, we obtain that $\mathcal {G}_{0}=C\ {\Delta }\sigma \ u$. Given that the stress drop Δ*σ*, though highly variable in earthquakes, shows no general dependence on *u*, and that *G* at rupture arrest scales with $\mathcal {G}_{0}$, it follows that fracture energy should scale linearly with slip, i.e.,

5 $$ G\propto u $$

to allow the arrest of earthquakes of all sizes. This preliminary result indicates that fracture energy in earthquakes is not necessarily related to an intrinsic material property such as material toughness, unless we assume that separate magnitude earthquakes occur on separate faults with arresting barriers of distinct properties at fault ends.

We discuss fracture energy in terms of frictional weakening, since both can be related as illustrated in Eq. 2. In high-velocity friction experiments, the observed abrupt dynamic weakening is clearly related to thermal decomposition, phase changes, breakdown reactions, etc. under intense frictional heating (Han et al. 2007; Di Toro et al. 2011).

Thermal pressurization of fluids trapped in the fault zone has also been invoked as a possible mechanism, and modeled under a series of assumptions, in particular about fault zone width and permeability (Rice 2006; Viesca and Garagash 2015). Under favorable circumstances, thermal pressurization may effectively preclude thermal decomposition in seismogenic crust faults by limiting coseismic temperature rise.

We remark that in high slip velocity experiments conducted in the presence of undrained fluid (water), a minor degree of pressurization is observed only in the later phases of slip (Violay et al. 2015). In both dry and wet experiments, decomposition weakening mechanisms appear to be triggered extremely early and efficiently (see description of weakening phases in Section 2), buffering the temperature rise and reducing the range of conditions where TP may be important on natural faults.

The experimental setup of Violay et al. (2015) allowed only to explore pressurization for a limited set of conditions: bare rock (no pressurized gouges were explored) and fixed fluid volumes. More efficient TP was predicted only through extrapolation of the experimental results to a different set of conditions. These suggested that an efficient TP would require that small volumes of fluids be trapped in a low permeability fault zone—such that a reduced heat capacity would maximize fluid temperature increase, and that diffusion may not buffer the pressure rise (for a complete discussion, see Violay et al. 2015). Otherwise, TP may become important mostly after large slip amounts (hence on mature faults generating large earthquakes, a point also suggested by modeling Viesca and Garagash 2015).

We will not develop here the physical interpretation of thermal decomposition weakening, nor endeavour in the design of a general constitutive relation for high-velocity friction. These will be the the topics of a separate dedicated study.

Instead, we analyze in detail the features of the experimental weakening curves and provide a general fit which is purely empirical, with the synthesis of a large number of experiments and their result in terms of frictional breakdown energy *G*_*f*_. We attempt to reconcile seismological studies and laboratory measurements of dissipation and friction.

It is striking that previous laboratory experiments (Wong 1982, and reference therein) repeatedly obtained markedly lower values of fracture energy (by at least three orders of magnitude) than those estimated for earthquakes from simple scaling relationships (Aki  1979), at least for intermediate to large magnitudes.

Here, the laboratory measurements show a remarkable compatibility with the seismological estimates of fracture energy. This is arguably due to the experimental conditions, which cover an extended range here to approach those of natural earthquakes. In particular, high slip velocity (>1 m/s), large amounts of cumulative slip (0.001–10 m), and intermediate normal stress conditions (10–30 MPa) are combined.

*G*_*f*_ can be obtained directly by integrating the experimentally measured shear stress curve *τ*(*u*)−*τ*(U) with respect to slip *u* according to Eq. 2. Since the earthquake estimates of *G* and the laboratory measures of *G*_*f*_ are conceptually and dimensionally equivalent quantities, we proceed to the comparison of our experimental data with both newly derived and previously published seismological estimates.

We discuss the similarities and the differences between *G* ^′^ and *G*_*f*_, and argue that dissipation on faults does include friction but also more general damage forms occurring off-fault (Andrews 2005). In recent years, it has been argued that an anelastic (off-fault plastic) damage zone forms adjacent to the fault, owing to the large stress concentration in the vicinity of the rupture tip (Reches 1999; Dor et al. 2006; Mitchell et al. 2011; Fondriest et al. 2015). In addition to the on-fault friction, which is measured in the experiments herein, during rupture propagation on natural faults the off-fault damage process may exert important controls on rupture, including reduction in rupture velocity and peak slip velocity (Andrews 2005), rupture arrest (Hok et al. 2010), influence on rupture directivity, and on the formation of short slip pulses (Xu et al. 2015). The width of the damage band generally increases with cumulative fault slip (Shipton et al. 2006b), increasing the dissipated energy in off-fault plastic strain, with the consequence that apparent fracture energy increases with rupture length. Damage can be induced by stress concentration in the vicinity of the propagating rupture tip (Andrews 2005). In addition, we discuss possible forms of dissipation arising from distributed deformation occurring in the accommodation of slip onto fault surfaces which are non-planar and present roughness at all scales.

## Dynamic weakening in high velocity rock friction experiments

Following Nielsen et al. (2015), we report the general trend of friction weakening, and the corresponding frictional fracture energy, for a representative sample of 28 experiments selected from a larger catalogue (experiments with low signal-to-noise ratio were chosen for a variety of conditions and lithologies, from more than 1000 experiments in total). All experiments were performed on a high-velocity, rotary shear apparatus (SHIVA) installed at INGV-Roma (Di Toro et al. 2010). The experiments were performed on cohesive, pre-cut rock samples representative of crustal seismic environments within the basement: silicate-rich rocks (microgabbro, Niemeijer et al. 2011; basalt, Violay et al. 2014) and calcite-rich rocks (Carrara marble with 99 % calcite, Violay et al. 2013); methods are described in detail in the references above. Experimental conditions varied, with imposed normal stress in the range 5–40 MPa, under velocity control with maximum sliding velocity 1–6.5 m/s, and slip accelerations (at the start of the experiments) of 3–6.5 m/s^2^. Samples were exposed to conditions of room humidity and temperature throughout the experiments (although during the high velocity slip intervals, temperatures in the vicinity of the slip surface increased in excess of 1000^∘^C under the effect of frictional heating). As observed in previous studies of friction at seismic slip velocity (≈1 m/s), frictional melting develops during the experiment for silicate-rich rocks (microgabbro and basalt, Tsutsumi and Shimamoto 1997; Hirose and Shimamoto 2005; Violay et al. 2014), but not on calcite-rich rocks (Han et al. 2007).

Dynamic weakening measured during high-velocity friction experiments under high normal stress shows substantial variations. However, when the experimental procedure is accurate enough to allow good signal-to-noise ratio and a high degree of repeatability, several systematic features are observed—for an example of high-velocity rotary friction (HVRF) experimental repeatability, see Violay et al. (2015). We will now analyze in detail the anatomy of the weakening curve in a couple of representative examples.

**Fig. 1:**
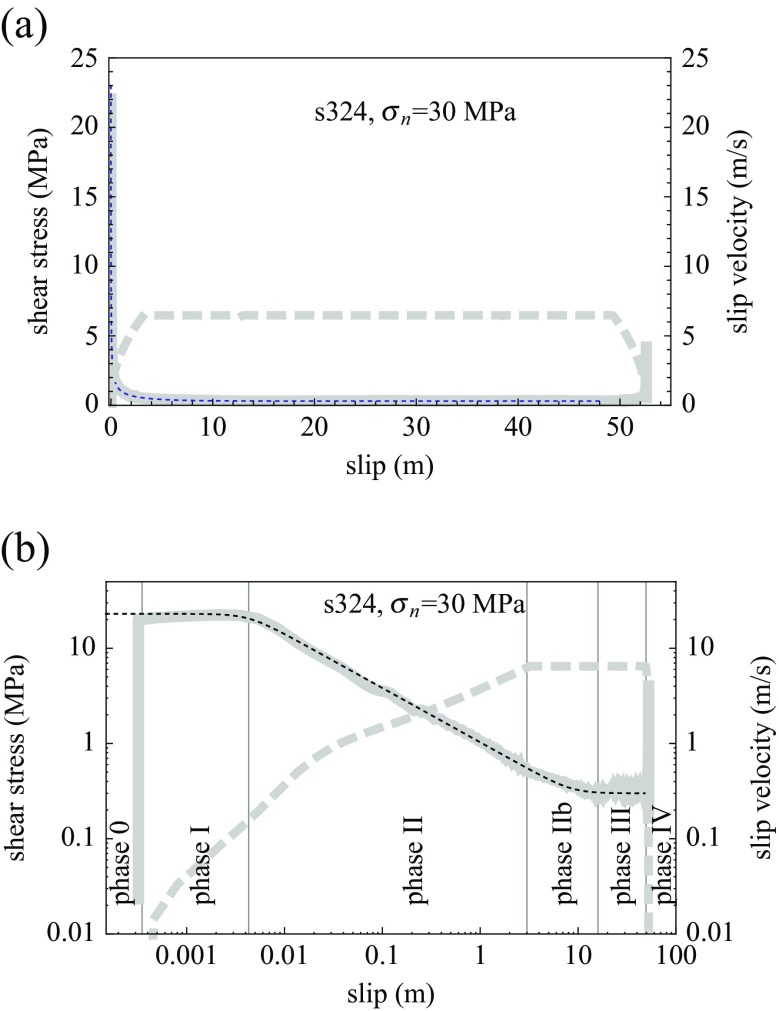
Shear stress (experimental weakening curve in *solid grey*), power law fit (*dashed black*), and slip velocity (*dashed grey*) for experiment S234, performed on Carrara marble at normal stress 30 MPa. **a** Linear plot. **b** Log-log scale plot (different phases are indicated as defined in text). The power law fit is according to Eq. 9, with parameters *u*_*w*_ = 0.0043 m, *τ*_*p*_ = 23 MPa, *τ*_*d*_ = 0.3 MPa, *α* = 0.57. In phase II–IIb, the weakening can be approximated by *τ* ∝ *u* ^−0.57^

As illustrated in Fig. 1b, upon rapid loading, shear stress rises linearly with strain as the sample is elastically loaded previous to the start of slip (phase 0). A short slip (phase I) then occurs under high, almost constant or slightly strain-hardening friction (compatible with Byerlee’s law with a friction coefficient >0.5). Phase I gives way very early (<1 cm of slip, which corresponds roughly to slip rates of the order of 10–20 cm/s under high imposed accelerations) to (phase II) when the high frictional power triggers efficient lubrication processes (Di Toro et al. 2011), and thus abrupt weakening is initiated. During all of phase II, friction drop versus slip follows a linear trend in a log-log diagram (Fig. 1b). Steady-state (phase III) in silicate-rich samples is achieved almost immediately after the target slip velocity has been reached (end of acceleration phase), where a low sliding friction value (<0.1) is maintained, with minor fluctuations, as long as slip rate is not modified. Note that in experiments under lower normal stress on gabbro, the steady-state is achieved much later during the steady-velocity sliding, and a second peak of strengthening is observed (Hirose and Shimamoto 2005). These differences are due to the larger normal stress, slip velocity, and the resulting larger frictional power dissipated in the experiments discussed here, whereby weakening is accelerated (Nielsen et al. 2010; Di Toro et al. 2011).

**Fig. 2:**
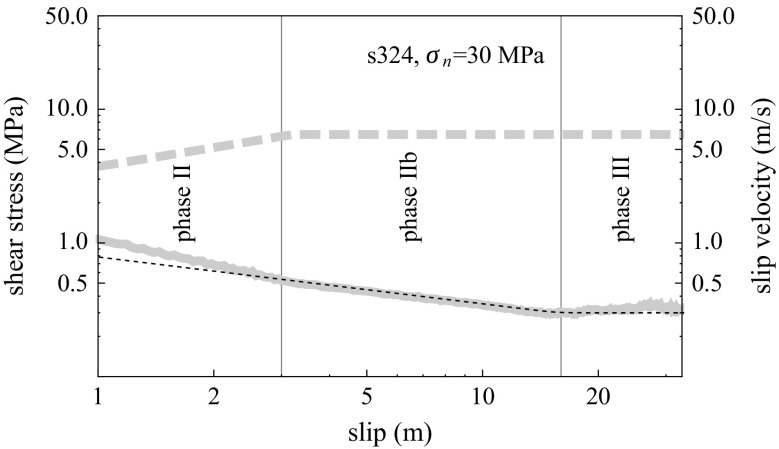
Detail of phase IIb of experiment S324 (same as Fig. 1). Phase IIb shows the continuation of weakening under constant slip rate; shear stress decay is best fit by *τ* ∝ *u* ^−0.35^ (*black dashed curve*) in phase IIb (as opposed to *τ* ∝ *u* ^−0.57^ in phase II)

In the case of calcite-rich samples, an intermediary (phase IIb) is observed during which a slight weakening continues even after the end of slip acceleration; in this case, phase III is reached only after several meters of slip. During phase IIb, friction versus slip still appears as log-log linear but with a reduced slope (Fig. 2).

Deceleration of slip (phase IV) is characterized by a rapid recovery of friction; in many experiments, the recovery reaches about 20 % of the peak stress. This value obviously depends on the imposed deceleration rate: friction is strongly velocity-weakening but not instantaneously related to slip velocity, so that a faster deceleration results in reduced recovery. A discussion on friction recovery is found in Del Gaudio et al. (2009); however, in that case, the deceleration was not imposed by the control system as in the experiments discussed here, but resulted from rapid dissipation of the moment of inertia in the rotary shear apparatus.

**Fig. 3:**
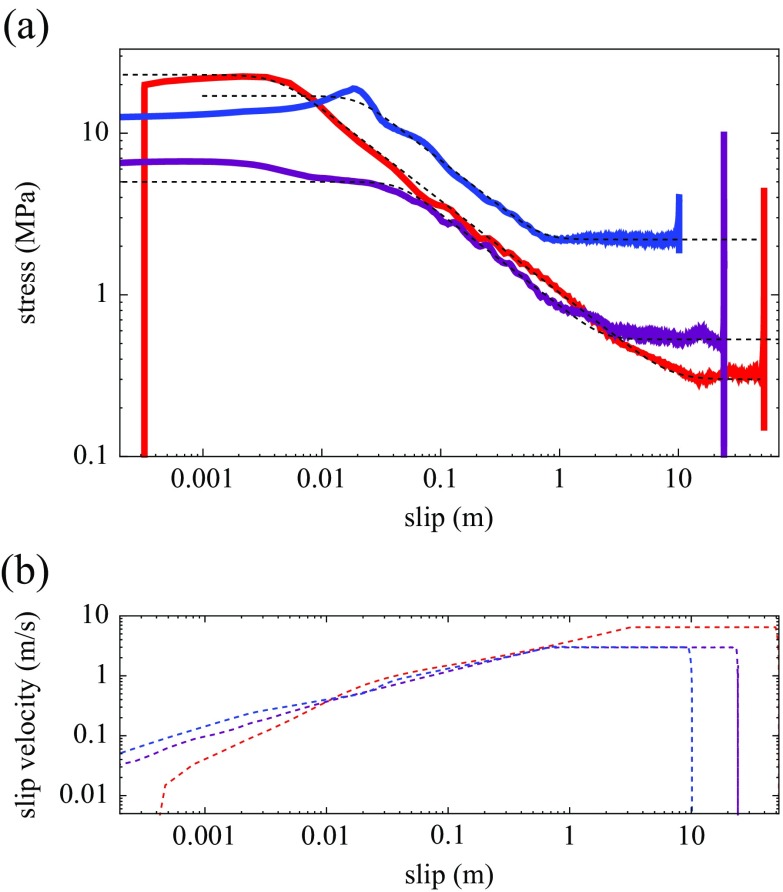
Comparison of experiments S324 (*red*) and S330 (*purple*), performed on calcite (*Carrara marble*), and S543 performed on gabbro (*blue*). Acceleration/deceleration is 6.5 m/s^2^ in all cases. **a** Shear stress as a function of slip; the *thin dashed lines* indicate the analytical fit according to Eq. 9, with parameters *u*_*w*_ = 0.0043 m, *τ*_*p*_ = 23 MPa, *τ*_*d*_ = 0.3 MPa, *α* = 0.57 (S324); *u*_*w*_ = 0.045 m, *τ*_*p*_ = 5 MPa, *τ*_*d*_ = 0.53 MPa, *α* = 0.57 (S330); *u*_*w*_ = 0.023 m, *τ*_*p*_ = 17 MPa, *τ*_*d*_ = 2.2 MPa, *α* = 0.57 (S543). Shear stress decay in phase II is always close to *τ* ∝ *u* ^−0.57^. **b** Slip velocity curves for the three experiments (same color code)

During the abrupt weakening phase, shear stress is best described by a power law of the form *τ* ∝ *u* ^−*α*^ where 0.5<*α* < 0.6 for phase II (*α* ≈ 0.35 for phase IIb). Given that the weakening phase is tapered at its beginning by an approximately constant peak value *τ*_*p*_ and at its end by the steady-state, dynamic sliding value *τ*_*d*_, the three different stages can be described by:

6 $$ u\ll u_{w},\ \ \tau\approx\tau_{p}  $$

7 $$ u_{w}<u<u_{w}(\tau_{p}/\tau_{ss}-1)^{1/\alpha},\ \ \tau\approx\tau_{p}\ \left( \frac{u}{u_{w}}\right)^{-\alpha}  $$

8 $$ u\gg u_{w}(\tau_{p}/\tau_{ss}-1)^{1/\alpha},\ \ \tau\approx\tau_{ss}  $$

where *u*_*w*_ is the slip value at which weakening is initiated; *τ* will have dropped to steady state value *τ*_*ss*_ when slip has reached *u* = *u*_*w*_(*τ*_*p*_/*τ*_*ss*_−1)^1/*α*^. An empirical fit for the entire frictional phases I–III (excluding phase IIb and the recovery phase IV) can be obtained in the form

9 $$ \tau=\left( \frac{{\tau_{p}^{n}}}{1+\left( \frac{u}{u_{w}}\right)^{\alpha\ n}}+\tau_{ss}^{n}\right)^{1/n}  $$

where *n* is an ad hoc integer introduced for convenience, to match the curvature of the shear stress function at start and end of the weakening phase (here we used *n* = 8). The fit corresponding to Eq. 9 is represented as a dashed curve along with the experimental data in Figs. 1, 2, and 3. The exponent 0.57 was obtained by least-squares minimization during phase II for all the experiments together; the tapering (*τ*_*p*_, *τ*_*ss*_) and the slip for onset of weakening (*u*_*w*_) are introduced for each individual curve by trial-and-error.

Under approximately constant slip acceleration $\dot {V}$, we shall have $V=\sqrt {2\ u\ \dot {V}}$. Assuming that weakening is triggered once a critical slip velocity *V*_*w*_ has been reached, then the corresponding slip is

10 $$ u_{w}\approx\frac{1}{2}\frac{{V_{w}^{2}}}{\dot{V}}.  $$

For experiment S324 (Fig. 1), we measure *V*_*w*_ ≈ 0.16 m/s and $\dot {V}\approx 3\ \text {{m\ s}}^{-2}$, resulting in *u*_*w*_ ≈ 0.0043 m. The exponent *α* is close to 0.5 in phase II, with a best fit value is *α* = 0.57. Using the rough approximation that *τ*∝1/*V* during the weakening phase, and noting that $V=\sqrt {2\ u\ \dot {V}}$ under constant acceleration, one would obtain $\tau \propto \frac {1}{\sqrt {2\ \dot {V}}}u^{-0.5}$, where the exponent value is fairly close to the experimental fit of *α* = 0.57 (this value holds for many experiments during phase II).

A comparison of two experiments performed on calcite under different maximum slip velocity and normal stress shows that phase II is very similar (Fig. 3); the change in the loading conditions mainly affects the values of *τ*_*p*_, *τ*_*d*_, and *u*_*w*_.

**Fig. 4:**
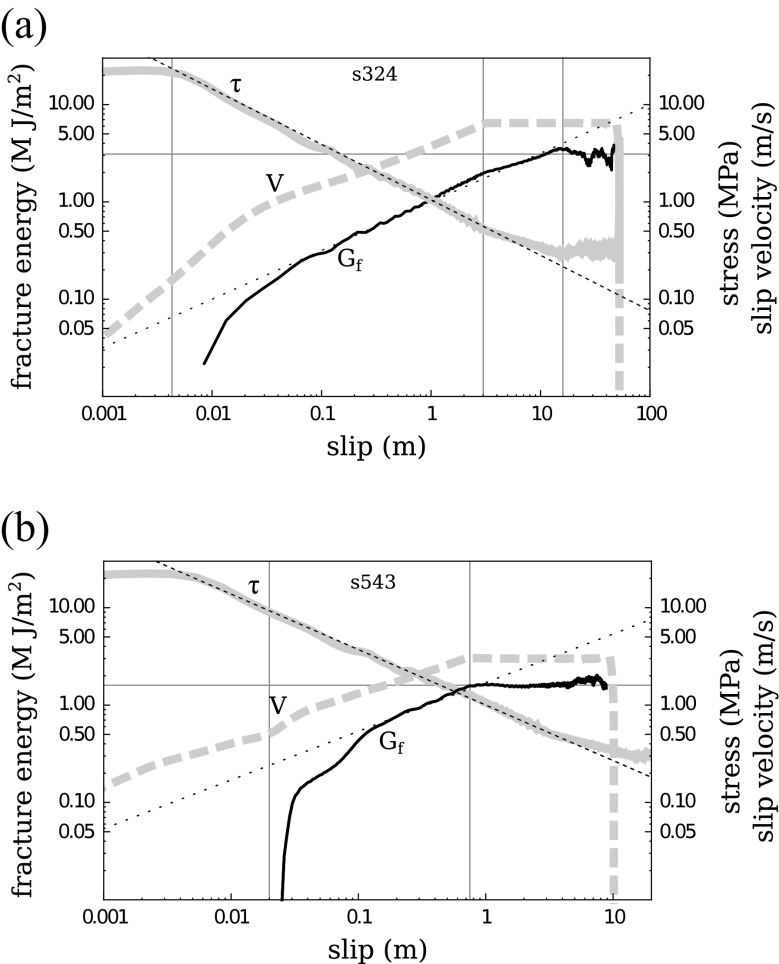
Frictional equivalent fracture energy *G*_*f*_ (*solid black curve*), obtained by integration of the data according to Eq. 2. Stress (*solid grey*) and velocity (*dashed grey*) are also represented. (*Dotted and dashed black lines* are indicative log slopes of −0.57 and +0.5, respectively). **a** Experiment S324 performed on calcite. **b** Experiment S543 performed on gabbro. Note that the gabbro experiment (**b**), reaches steady-state much earlier because of frictional melting, thus *G*_*f*_ saturates much earlier, than the calcite experiments (slip about 1m as opposed to 10 m). See text for further details

In case of silicate-rich rocks, no phase IIb is observed, and steady-state is reached much earlier, as soon as the slip acceleration phase is over. Frictional melt and extrusion of melt out of the slipping zone takes place in silicate-rich rocks with relatively rapid (*ν* ≈ 1 mm/s or more under our experimental conditions) shortening of the sample. As discussed by (Nielsen et al. 2008, 2010), the melting front advances into the sample catching-up with the thermal diffusion, creating a thermal boundary layer of finite thickness in such a way that a steady-state is rapidly reached. The steady-state thickness of the boundary layer is *b* = *κ*/*ν* where *κ* is the thermal diffusivity and *ν* is the melting front velocity (or sample shortening half-velocity). Dimensional arguments indicate that steady-state will be reached within a time *t* ≈ 2*κ*/*ν* ^2^ (less than 1 s and less than 1 m slip at our experimental conditions). With increased frictional power dissipation (hence with increased slip velocity, acceleration, and normal stress), sample shortening velocity *ν* is faster and steady-state is achieved in reduced time and slip amounts. One example for gabbro (experiment S543) is shown in Figs. 3 and 4; the main differences with calcite experiment of Fig. 1 are the slight slip-hardening phase before weakening, the absence of phase IIb and the faster achievement of steady-state. Besides these differences, the same general fit (Eq. 9) and exponent as for the calcite example applies to cases of frictional melting.

In conclusion, the sliding shear stress during the weakening interval (phase II–IIb) is reasonably well described by a power law in slip, tapered by peak stress and steady-state dynamic stress on either end. The steady-state is achieved at shorter slip for melt experiments, where it is further anticipated by higher normal stress and slip rate. However, steady state is not anticipated by normal stress increase in calcite experiments, where no melting occurs, although this impression is conveyed when inspecting the shear stress curves on linear axes plots due to the higher peak stress value.

Measures of a slip-weakening distance *D*_*c*_ have been attempted previously in high velocity friction experiments; *D*_*c*_ was defined as the distance over which *τ*−*τ*_*ss*_ drops down to 0.05 (*τ*_*p*_−*τ*_*ss*_) (Han et al. 2010). It has been argued in the case of frictional melt (Nielsen et al. 2008, 2010) that the apparent slip-weakening distance scales as $D_{c}\propto 1/(V {\sigma _{n}^{2}})$ (inverse of velocity and inverse normal stress square). However, in the light of the fact that, under different experimental conditions, the weakening part of the curves collapse into a single powerlaw, where changes mostly affect initial (*τ*_*p*_) and steady-state (*τ*_*p*_) values, the significance of *D*_*c*_ defined as a characteristic distance becomes less clear.

## Fracture energy and frictional weakening

Using Eqs. 6 and 7, we can obtain a theoretical prediction for fracture energy $G_{f}(u)=u_{w}\tau _{p}\left (\frac {\alpha }{\alpha -1}+(\frac {u}{u_{w}})^{-\alpha }+\frac {1}{1-\alpha }(\frac {u}{u_{w}})^{1-\alpha }\right )$ by integrating the frictional weakening according to Eq. 2. Given that *α* < 1, in the case that *u* ≫ *u*_*w*_, we may neglect first two terms to obtain:

11 $$ G_{f}(u)\approx\frac{1}{1-\alpha}\ u_{w}\tau_{p}\left( \frac{u}{u_{w}}\right)^{1-\alpha},\ \text{for}\ u<u_{w}(\tau_{p}/\tau_{ss}-1)^{1/\alpha}  $$

12 $$\begin{array}{@{}rcl@{}} &&G_{f}(u)=\text{const.}\approx\frac{1}{1-\alpha}\ u_{w}\tau_{p}\left( \tau_{p}/\tau_{ss}-1\right)^{\frac{1-\alpha}{\alpha}},\ \\ &&\quad\text{for}\ u\ge u_{w}(\tau_{p}/\tau_{ss}-1)^{1/\alpha} \end{array} $$

the second equation indicates that *G*_*f*_ saturates after reaching the steady-state with *τ*_*d*_ at *u* = *u*_*w*_(*τ*_*p*_/*τ*_*ss*_−1)^1/*α*^.

In a similar fashion, we may compute the discrete summation equivalent to Eq. 2 using *τ* and *u* measured in the experiments, to obtain an experimental curve *G*_*f*_(*u*).

**Fig. 5:**
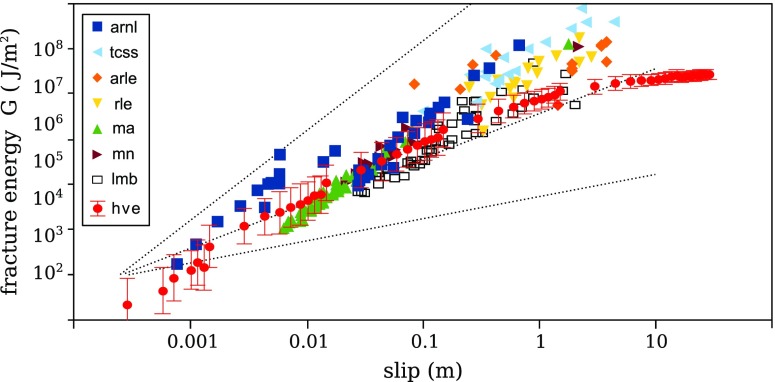
Experimental (*G*_*f*_, in *red*) and seismological (*G* ^′^, other colors) estimates of fracture energy under coseismic slip conditions. *Dashed lines* indicate exponent 0.5, 1, and 2 for reference. *Red disks* correspond to values of *G*_*f*_ averaged for 28 different experiments, at various slip amounts. arnl: Northridge aftershocks (Abercrombie and Rice 2005); arle: large earthquakes (Abercrombie and Rice 2005); tcss: numerical simulations (Tinti E. et al. 2005); rle: large earthquakes (Rice 2006); ma: L’Aquila (Malagnini et al. 1994); mn: Northridge sequence (Malagnini et al. 1994); lmb: Tocopilla, Chile (Lancieri et al. 2012); hve: high velocity friction experiments

The latter is represented as a solid black curve in Fig. 4 for two examples (calcite S324 and gabbro S543), and an average *G*_*f*_ for the 28 different experiments mentioned in the previous section is shown in (Fig. 5). Note that the friction recovery phase during the deceleration takes place within a very small slip amount, so that the frictional energy related to recovery is negligible and is not taken into account here in the definition of *G*_*f*_. A consequence is that *G*_*f*_(*u*) obtained at any partial slip value *u* < *u*_*final*_ can be considered as a reliable prediction of the final *G*_*f*_ that would have resulted for an experiment arrested earlier at *u*_*final*_ = *u* (see Appendix for details on the computation of experimental *G*_*f*_). On the other hand, we anticipate that the presence of a strong recovery will introduce a non negligible difference between *G*_*f*_ and the seismological estimate *G* ^′^ which is obtained under the assumption of no undershoot (no recovery), as discussed further below.

## Comparison of *G*_*f*_ with values estimated from seismological data

The scaling shown by Eq. 7 is, in some aspects, in agreement with independent estimates of earthquake fracture energy. Equating the fracture energy to the difference between the available strain energy (in excess of the minimum sliding friction) and the radiated energy (as quantified from kinetic energy in the far-field), Abercrombie and Rice (2005) (hereafter AR) proposed an estimate of *G* ^′^, the apparent fracture energy, noting that *G* ^′^ and the actual *G* will coincide if the final stress is equal to the minimum sliding shear stress (no undershoot, i.e., final stress higher than the sliding stress, neither overshoot, i.e., final stress lower than the sliding stress). Based on the best-fit of a number of earthquake data AR proposed the empirical relation:

13 $$ G^{\prime}=5.25\;10^{6}\;u^{1.28}  $$

(we use *G* ^′^ for fracture energy values resulting from seismological estimates, following AR, as opposed to *G*_*f*_ resulting from experimental measurements). In addition, Tinti E. et al. (2005) proposed *G*_*k*_ ∝ *u* ^1.81^. Their estimate was obtained by imposing the slip retrieved from kinematic inversion as a boundary condition in dynamic simulations, and the resulting stress evolution was computed.

In Fig. 5, *G* ^′^ from a literature compilation (Nielsen et al. 2015) and the average *G*_*f*_ value for all 28 high-velocity experiments are shown together. *G*_*f*_ and *G* ^′^ are comparable for slips of about *u* = 1 cm (*G* ≈ 10^4^ J/*m* ^2^), as reported by Nielsen et al. (2015), with the addition here of the Tocopilla earthquake sequence (Lancieri et al. 2012). *G* ^′^ and *G*_*f*_ both increase with slip up to about 10^6^ J/m^2^, however, while *G*_*f*_ saturates at *u* ≈ 10 m, it appears that *G* ^′^ continues to increase, with the result that for some of the reported earthquake sequences *G* ^′^ is substantially larger that *G*_*f*_ at large magnitudes (*u*≥10m). The exponent in *G*_*f*_ (1−*α* ≈ 0.5 ) is lower than in *G* ^′^ (1−*α* ≈ 1.28 as reported by AR or slightly more for the earthquake sequences reported here). We discuss further below the possible causes of such discrepancy.

Assuming that *G* ^′^(*u*) and shear stress *τ*(*u*) are related through Eq. 2, and that *G* ^′^ has the form of Eq. 13, AR took the derivative of both with respect to *u* and after some algebra obtained a stress evolution of the form:

14 $$ \tau(u)=C_{0}-4.8\ 10^{6}\ u^{0.28}.  $$

*C*_0_ can then be assimilated to the peak shear stress *τ*_*p*_ at *u* = 0. If this form were used with very low values of *τ*_*p*_ (e.g., 5 MPa) and relatively high values of *u* (e.g., 10 m), although it is not a very likely combination of parameters for earthquake source, it may result in the unphysical feature of a negative friction. On the other hand, if the exponent of 1.28 in (Eq. 13) is replaced by any positive value smaller than 1 (for example 0.5), then retrieving *τ* from expression (2) results in a quite different expression for shear stress evolution, of the form *τ* = *C* + *B* *u* ^−0.5^ (where *C* and *B* are constants to be determined by limit conditions), which can produce a weakening law without incurring into negative friction. (By setting *C* = 0 and $B=\tau _{p}\sqrt {u_{w}}$, we do obtain a solution of the type of Eq. 7). Thus, a value greater than 1 in the exponent in Eq. 13 resulting from seismological estimates produces an unphysical friction law if we assume that *G* ^′^ is related to frictional dissipation alone.

On the other hand, assuming that a single powerlaw scaling such as Eq. 13, and a single associated dissipation mechanisms, would represent earthquake mechanics over a seven magnitude-range or more is an oversimplification. It has been pointed out before (Rice 2006; Viesca and Garagash 2015) that Eq. 13 fails to capture the trend for earthquakes in the larger magnitude range. As magnitude increases, transitions in the weakening mechanisms and in the rupture mode (from crack-like to pulse-like, Viesca and Garagash (2015)) may be possible causes of the observed changes in *G* ^′^(*u*). In addition, uncertainty in *G* ^′^ for large earthquakes is due to the difficulty of including large near-field radiation and/or the use of finite-source kinematic inversions with non-unique solutions. Indeed, a large scatter in fracture energy *G* ^′^ is observed at large magnitudes (Fig. 5), with the lower end members (*lmb*, Tocopilla sequence) being broadly compatible with the experimental measurements of *G*_*f*_.

The above comments suggest either (1) that *G* ^′^ estimates may be affected by uncertainty or bias in the large magnitude; or (2) that *G* ^′^ represents a measure of other dissipation processes that friction alone. In the latter case, friction needs to be redefined in an equivalent, broader sense suitable for the scale of earthquake faults. A transition from friction-dominated dissipation to diffusive-dominated processes may occur in larger earthquakes. For example, thermal pressurization mechanisms may become more effective at larger slip; diffuse anelastic co-seismic strain may affect a wider fault zone on mature faults and become important in the energy budget of rupture.

In the following sections (Sections 5–9), we consider different sources of mismatch between *G* ^′^ and *G*_*f*_ at large magnitudes, due to either a bias in the seismological estimates *G* ^′^, or to the limitations of using experimental measures of friction to account for large earthquake dissipation.

First, we will consider whether a more complex loading history than that used in the experiments (constant acceleration ramp) may radically alter the *G*_*f*_ trend and whether it may approach that of *G* ^′^ under realistic conditions (Section 5). Subsequently, we discuss the effect of normal stress increase in the experiments, showing that it has no significant influence on the fracture energy, except for slip <10 cm (Section 6). Then, we discuss whether a bias due to undershoot in the *G* ^′^ estimates may explain the difference with *G*_*f*_ (Section 7). Finally, we consider possibilities of *G* ^′^ being the signature of other dissipation processes than friction: off-fault plasticity (Section 8) and non-planar fault topography, discussing possible scaling of *G* ^′^ with *u* due to fault non-planarity (Section 9).

## Possible outcomes of a different loading history

As noted in Section 2, using the rough approximation that during the weakening phase friction is inversely proportional to slip velocity such that *τ*∝1/*V*, and noting that $V=\sqrt {2\ u\ \dot {V}}$ under constant acceleration, one would obtain $\tau \propto \frac {1}{\sqrt {2\ \dot {V}}}u^{-0.5}$, a power law of slip with an exponent fairly close to the experimental fit *α* = 0.57. We remark that it is a limitation of the experiments that a constant acceleration $\dot {V}$ is used, followed by a constant “target” velocity. Given the close link between *τ*(*t*) and *V*(*t*), it is expected that the scaling *τ* versus *u* may differ to some extent if the loading time history was altered. Nonetheless, most dynamic rupture models produce a very abrupt acceleration phase in the vicinity of the rupture tip (corresponding more or less to the frictional weakening phase), which we assume here can be reasonably linearized by assuming constant $\dot {V}$. More marked differences in the slip velocity are expected after the acceleration phase. Indeed, models (Kostrov 1974; Andrews 1976; Broberg 1999) predict a peak velocity near the end of the weakening phase followed by a relatively rapid decay in *V*, whereas the current experiments impose a constant slip rate; however, this later evolution would not alter the fracture energy which mostly results from the stress evolution within the weakening phase only.

If we now assume that the approximation *τ*∝1/*V* holds but drop the assumption of constant acceleration and set *τ* = *τ*_*p*_−*C*_1_ *u* ^*β*−1^, where—compatibly with *G* ^′^ estimates and Eq. 2—we have *C*_1_ ≈ 4.8 10^6^ and *β*−1 ≈ 0.28. Using an indicative value *τ*_*p*_ = 100 MPa, this would require that slip velocity during the weakening phase follows $V\propto \frac {10^{-6}}{100-4.8\ u^{0.28}}$. It is clear that in this case, velocity is increasing unrealistically slowly with increasing slip, as this contradicts (1) the abrupt acceleration resulting from the elastodynamic solution systematically observed in rupture propagation models and (2) the observation that wave radiation is mostly generated in the vicinity of the rupture front, which again supposes an abrupt acceleration of slip.

As a consequence, we think it is unlikely that the difference between *G* ^′^ and *G* may be reconciled by assuming a different slip velocity profile, as the latter would hardly be compatible with fundamental results of elastodynamics and with seismological observations.

## Influence of normal stress

We now discuss whether the normal stress does have an influence on fracture energy, and whether it may justify the difference between the experimental measures and the seismological estimates of fracture energy. While the normal stress in the experiments discussed here is in the range 10–40 MPa, we expect it to be higher on natural faults at seismogenic depths. Normal stress resolved on the fault will depend on the deviatoric stress and the type of faulting (Malagnini et al. 2010), but it will on average be of the same order as the lithostatic load. At the base of the seismogenic crust (≈10–15 km), the lithostatic load can be up to ≈300 MPa assuming no pore pressure, ≈200 MPa under hydrostatic pressure and considerably less in case of overpressure. The arithmetic average in the ten topmost kilometers may therefore be up to about 150 MPa.

Normal stress influences the peak and the steady-state shear stress, and the apparent weakening distance (Nielsen et al. 2010). However, previous theoretical modeling of frictional melt and related experimental data obtained in rotary shear experiment showed no systematic variation of fracture energy with normal stress. Here, we confirm these preliminary results by extending them to a wider range of normal stress and to either cases where frictional melt is taking place (gabbro, basalt) or not (calcite experiments).

**Fig. 6:**
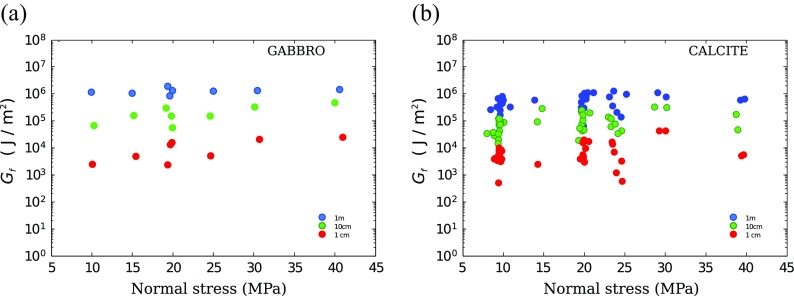
Measure of fracture energy for experiments performed under different normal stress (in the range ∼10 to ∼40 MPa) and three different slip amounts (*red* = 1 cm, *green* = 10 cm, *blue* = 1 m). No significant trend is observed with normal stress. The increase with slip is replicates the observation of Figs. 4 and 5. **a** Gabbro experiments; **b** calcite experiments

Figure 6 shows a collection of fracture energy measured under different normal stress and at three different values of slip. For the case of gabbro, at slip of 1 and 10 cm, there appears to be an order magnitude increase in *G*_*f*_ as normal stress varies from 10 to 40 MPa. However, it is to note that at 1 and 10 cm experiments indicate a degree of variability of the same order of magnitude (experiments at 20 MPa were repeated). As a consequence, such trend is not significantly above the experimental error. When the weakening is about to be complete (1 m slip), the data variability is reduced and no systematic trend is observed in *G*_*f*_ with normal stress. The data for calcite, on the other hand, do not show any significant trend above the variability.

## Systematic bias due to under- or overshoot

**Fig. 7:**
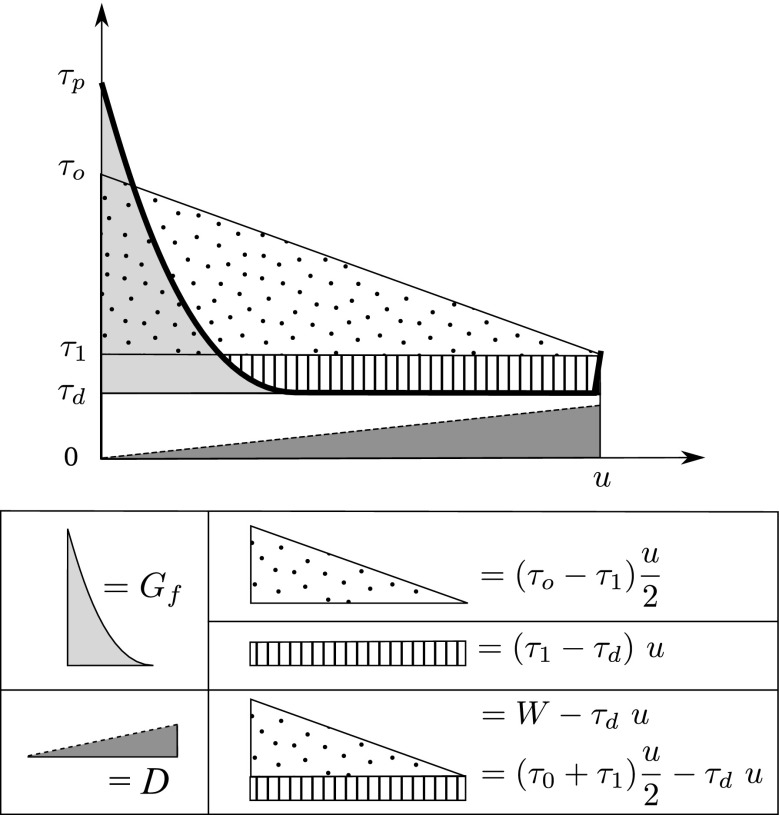
Schematic energy partition as a function of slip and initial, dynamic, and final stress. The total area below the diagonal line connecting (0, *τ*_0_) and (*u*, *τ*_1_) is the available elastic strain energy *W*. Frictional dissipation is the total area below the shear stress curve (*thick black curve*). *Light grey area* indicates the fracture energy due to friction, *G*_*f*_. The *hatched rectangular area* indicates the energy error incurred by neglecting *τ*_1_−*τ*_*d*_. The *dark grey triangle* below the *dashed line* (named *D*) indicates a general dissipation process (*not friction*) increasing with slip (*in this case linearly*). See text for futher details

Seismological estimates of *G* ^′^ are based on the assumption that there is no under- or overshoot in earthquakes (i.e., the assumption that final stress *τ*_1_ is equal to the minimum dynamic sliding stress *τ*_*d*_, see Fig. 7). Some consequences of such assumption are discussed in AR and (Viesca and Garagash 2015). Here, we explore the possibility the same assumption could cause the hiatus between the experimental and seismological fracture energies.

Because only the static (final) stress drop Δ*σ* = *τ*_0_−*τ*_1_ (*τ*_0_ is initial stress, see Fig. 7) can be estimated through seismology, but not the absolute stress or the initial, final and dynamic stress values, the estimate from earthquake data is $G^{\prime }=(\tau _{0}-\tau _{1})\frac {u}{2}-\frac {\mu ^{\prime }E_{s}}{M_{o}}\ u$ (under the assumption *τ*_1_ = *τ*_*d*_) based on average slip, stress drop, radiated energy *E*_*s*_, and seismic moment *M*_*o*_. Recalling the energy balance in the earthquake process already discussed elsewhere (Scholz 1990; Rivera and Kanamori 2005; Abercrombie and Rice 2005) and illustrating schematically stress and frictional work flow as of Fig. 7, we can write:

15 $$\begin{array}{@{}rcl@{}} G &=& W-\frac{E_{s}}{A}\ ,\\ &=& (\tau_{0}+\tau_{1})\ \frac{u}{2}-\tau_{d}\ u-\frac{E_{s}}{A}\ ,\\ &=& (\tau_{0}+\tau_{1})\ \frac{u}{2}-\tau_{d}\ u-\frac{\mu^{\prime}\ E_{s}}{M_{o}}\ u\ . \end{array} $$

(where *W* is the work of the elastic strain release, *μ* ^′^ is the shear stiffness, and *A* is the area of the fault). As a consequence *G* = *G* ^′^+(*τ*_1_−*τ*_*d*_) *u*. If the actual *G* was smaller than the *G* ^′^ approximation, and possibly in agreement with the experimental measure *G*_*f*_, this would imply that *τ*_1_ < *τ*_*d*_ (overshoot). However, only a weak overshoot is generated by crack-like ruptures under favorable circumstances (Madariaga 1976). On the other hand, pulse-like ruptures which provide a better fit to large eathquakes kinematic inversions (Heaton 1990), systematically generate undershoot (Nielsen and Madariaga 2003).

As a consequence, the assumption that *τ*_*d*_ ≈ *τ*_1_ for *G* ^′^ estimates is most likely to underestimate the actual fracture energy than otherwise. Therefore, this cannot reconcile the difference *G*_*f*_ < *G* ^′^ at large magnitudes.

## Off-fault energy sinks

As noted by Shipton et al. (2006a), the effective *G* ^′^ observed at the seismological scale should implicitly incorporate energy sinks other than frictional dissipation alone. On a planar fault, the stress level off-fault is sufficiently high to induce anelastic damage on a thickness increasing with length propagation, as initially proposed by Poliakov (2002). In a model by Andrews (2005), the dissipation due to plastic strain results in *G* scaling with slip so that *G*∝ *u* ^1.0^, an exponent which is also compatible with the barrier toughness model for the arrest of crack growth (discussed in Eqs. 3, 4, and 5). The exponent 1.0 is intermediate between that estimated from frictional dissipation and that estimated from seismological data.

Indicatively, a trend representing a general dissipation growing with slip is shown in Fig. 7 as the grey area the bottom of the diagram (we represent a case with *D* ∝ *u* ^1.0^ compatibly with models of off-fault plastic dissipation, but more general trends may be speculated, see section below on non-planar fault surfaces). This ulterior energy sink *D* has to be subtracted from the radiated energy. The balance of energy Eq. 15 then becomes:

16 $$\begin{array}{@{}rcl@{}} G+D &=& W-\tau_{d}\ u-\frac{E_{s}}{A}\ ,\\ &=& (\tau_{0}+\tau_{1})\ \frac{u}{2}-\tau_{d}\ u-\frac{\mu\ E_{s}}{M_{o}}\ u\ . \end{array} $$

The above shorthand form, where values are averaged on the fault rupture area *A*, can be equated to the more general integral form (Kostrov 1974; Rivera and Kanamori 2005):

17 $$\begin{array}{@{}rcl@{}} E_{s} &=& \frac{1}{2}{\int}_{A}(\tau_{1}+\tau_{0})\ u\ dA-{\int}_{A}2\ \gamma\ dS\\ &&-{\int}_{t0}^{t_{1}}dt{\int}_{A}\tau\ \dot{u}\ dA \\ &=& \ \frac{1}{2}{\int}_{A}(\tau_{1}+\tau_{0})\ u\ dA \\ &&- {\int}_{t0}^{t_{1}}dt{\int}_{A}\tau_{d}\ \dot{u}\ dA\-{\int}_{A}2\ \gamma\ dA\\ &&-{\int}_{t0}^{t_{1}}dt{\int}_{A}(\tau-\tau_{d})\ \dot{u}\ dA \end{array} $$

(where, for simplicity, we assumed that slip *u* is in a fixed direction (no rake rotation) to avoid vectorial notation). Here, we may equate $W=\frac {1}{2\ A}{\int }_{A}(\tau _{1}+\tau _{0})\ u\ dA$, $G=G_{f}=\frac {1}{A}{\int }_{t0}^{t_{1}}dt{\int }_{A}(\tau -\tau _{d})\ \dot {u}\ dA$ and $\tau _{d}\ u=\frac {1}{A}{\int }_{t0}^{t_{1}}dt{\int }_{A}\tau _{d}\ \dot {u}\ dA$. The remaining term ${\int }_{A}2\ \gamma \ dA$, was introduced to account for the energy sink in the singular stress terms ahead of the crack tip, considering the case where the process zone at the crack tip is vanishingly small. If we assume that for earthquakes there is no singularity strictly speaking and the process zone is finite, we may instead equate the *γ* term to diffuse off-fault dissipation in the vicinity of the crack tip, and ideally equate $D=\frac {1}{A}{\int }_{A}2\ \gamma \ dA$.

In addition, natural fault surfaces are rough at many different scales and the slipping zone is often made of non-cohesive rock (gouge), whereas experiments are performed on smooth, machined surfaces (wavelength <10^−3^ m) and, often, on cohesive rocks. We discuss possible effects of roughness and thickness of damage zone in the next section.

## Non-planar fault surfaces

The non-planarity of natural faults creates additional stress and strain during slip (Chester and Chester 2000; Sagy and Brodsky 2009; Griffith et al. 2010). If sufficiently strong, such stress may result in asperity abrasion (especially at small wavelengths) and off-fault damage which will introduce energy sinks in addition to that of friction on the sliding surface itself (Dunham et al. 2011). Moreover, in the presence of fault gouge (non-cohesive rock), typical at depths <6–7 km, a relevant portion of energy may be dissipated during the slip-localizing process. An example of 600 m wide damage structure associated to ancient seismic faulting at depth, has been reported in detail by Smith et al. (2013). Since G’ is obtained by estimating the amount of dissipation with respect to strain energy and radiated energy, it will implicitly incorporate the sum of all dissipative processes due to rupture propagation and fault slip, necessarily larger or equal to the laboratory, friction-only related measurements.

With increasing fault slip, the size of the topographical asperities which are removed, the wavelength of fault bends which are involved, and the thickness of the damage zone increases. As a consequence, the apparent G’ from seismological estimates, implicitly accounting for the effects of off-fault damage, may be larger than *G*_*f*_ and have a different slip dependence than the experimentally measured *G*_*f*_, which accounts for small-scale dissipation by friction alone.

**Fig. 8:**
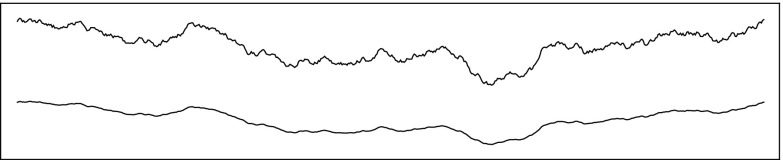
Comparison of self-affine randomly generated topographies with Hurst exponent *H* = 0.8 (*top curve*) within the range of values observed on fossil seismic faults exhumed from depths of ≈9 km (Bistacchi et al. 2011) and *H* = 1.14 (*bottom curve*). The larger *H* = 1.14 value, implying relatively larger fault topography at larger scales, but relatively smooth fault at smaller scales, would generate a strain energy per unit fault area which scales as *G* ^′^∝u^1.28^ as estimated by AR. The self-similar case corresponding to *H* = 1 (*not shown*) produces an intermediate roughness

Natural fault surface roughness can be described by a distribution with power spectrum *P*(*k*) = *k* ^−1−2*H*^ where *k* is wavenumber and *H* is the Hurst (or Hausdorff) exponent (Schmittbuhl et al. 1993; Bistacchi et al. 2011; Candela et al. 2012). Examples of synthetic topographic profiles are shown in Fig. 8 with *H* = 0.8 (and indicatively, though unrealistic, with *H* = 1.14). If a fraction of the elastic strain work is dissipated while the asperities are cyclically strained, such process may yield a dissipation increasing as a power of slip due to the increasingly large scale roughness involved.

In the following paragraphs, we provide estimated scaling of strain and dissipation induced by slip on a non-planar fault. Although a number of simplifying assumptions are made, the dimensional arguments and the resulting scaling should not be altered by the assumptions. Since the estimates rely on a model of infinitesimal strain and elastic stress, we note that our approximate model should produce an upper bound estimate. In future studies, a verification may be attempted by using numerical tools which possibly allow for finite strain analysis, a Lagrangian approach, and the implementation of anelastic strain.

We start our analysis using a simple sinusoidal topography *y* = *h* sin(2*π* *x*/*λ*) for the fault surface. Assuming that the fault surface itself is lubricated, elastic stress and strain induced by topography in the fault vicinity can be computed using the well-known analytical solution based on Flamant problem, under the assumption that amplitude *h* is small with respect to the wavelength *λ* (see for example Saucier et al. (1992)).

As slip increases on the fault, we may assume that a finite fraction of the elastic work spent in the cyclic compression and decompression of the sinusoidal bends is dissipated in anelastic strain and permanent damage. Damage in the vicinity of natural faults is consistently observed, and in some instances, it has been shown to correlate with the fault bends (Griffith et al. 2010).

### Fault with a single wavelength in topography

**Fig. 9:**
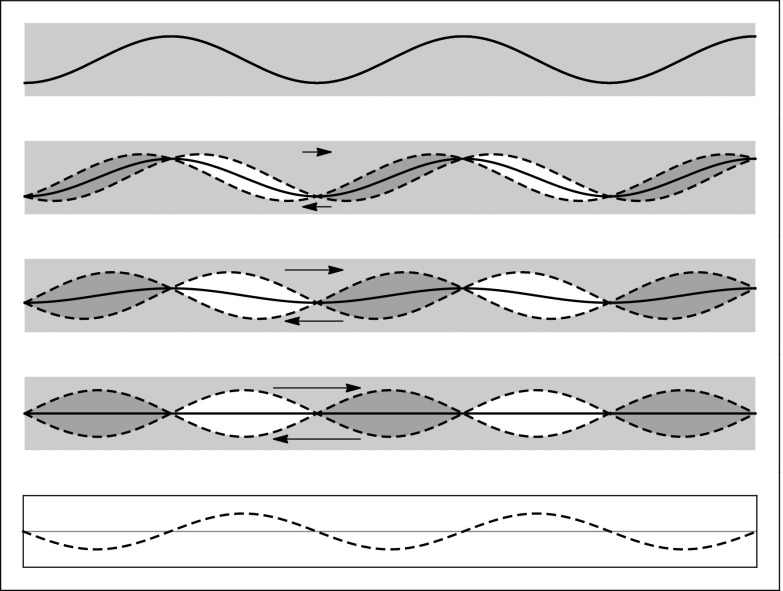
Wavy fault (*black thick curve*) after offsets of $\frac {1}{5}, \frac {2}{5}$, and $\frac {1}{2}$; *λ*. *Dashed curves* indicate the virtual boundaries of top and bottom sides if they were offset without strain (virtual overlap and underlap). At $u=\frac {1}{2}\lambda $, the single wavelength topography has flattened-out. For reference, the normal stress corresponding to $u=\frac {1}{2}\lambda $ is shown (*bottom dotted curve*)

We consider two ideal models of dissipation due to strain on a non-planar fault. The first model assumes that the stress distribution around then fault can be approximated by an elastic model, illustrated in Fig. 9, but that a portion of elastic strain energy is dissipated through each deformation cycle. The second model assumes that asperities are abraded by irreversible anelastic strain occurring in a thickness which is approximately that of fault topography elevation *h*(*λ*) at the wavelength *λ* ≈ *u*. Neither model is producing an exponent in dissipation compatible with the seismological estimates. To avoid the assumptions and approximations used in this analysis, a full numerical simulation could be performed which is beyond the scope of this study, for example, using methods illustrated by Dunham et al. (2011).

### Elastic stress and strain energy in cyclic deformation

We first discuss the elastic stress perturbation resulting from slip on a wavy fault of simple topography represented by *y* = *h*sin(2*π* *x*/*λ*). As discussed in Appendix, when the fault slips an amount *u* it will undergo *n* = *u*/*λ* strain cycles, resulting in an absolute value of elastic strain work which scales as w∝*u* (*h* ^2^/*λ* ^2^ + *A* *h*/*λ*) where *A* is a dimensionless ratio of prestress versus elastic stiffness (see Appendix). Assuming that the topographical elevation *h* can be related to wavelength such that

18 $$ h=\phi \ \lambda^{H}, $$

where *ϕ* is a normalizing factor, we may write w∝ *u* *ϕ* ^2^ *λ* ^2*H*−2^ + *u* *ϕ* *A* *λ* ^*H*−1^. For fault slip *u* ≈ *λ*, we obtain:

19 $$ \mathrm{w}\propto\ \phi^{2}\ \lambda^{2H-1}+A\ \phi\ \lambda^{H}  $$

which, given the dimensions of *ϕ* as of Eq. 18, yields correct dimensions of J *m* ^−2^.

For slip *u* on the fault, strain due to non-planarity will involve predominantly wavelengths *λ* ≈ *u* and below.

Although Eq. 19 is derived from the description of a purely elastic stress/strain relation, we may assume that if during each deformation cycle a fraction of work is dissipated through visco-elastic or plastic processes, the scaling may remain close to that of Eq. 19. Hence, we may consider w as an upper-bound estimate of the roughness-induced dissipation, and assuming that the strain is dominated by the upper wavelength *λ* ≈ *u*, we find

20 $$ G_{r}\propto u^{2H-1}+A\ u^{H},  $$

which may contribute to the apparent fracture energy *G* ^′^ in the earthquake balance. Using an indicative value *H* = 0.8, we obtain a scaling exponent of about 0.6 (if *A*≪1 in Eq. 20) or 0.8 (if *A*≫1). In both cases, the exponent is quite lower than the seismological estimates. Considering the effect of smaller wavelengths in the above computation, though not developed here, leads to similar results.

### Finite brittle or ductile strain

**Fig. 10:**
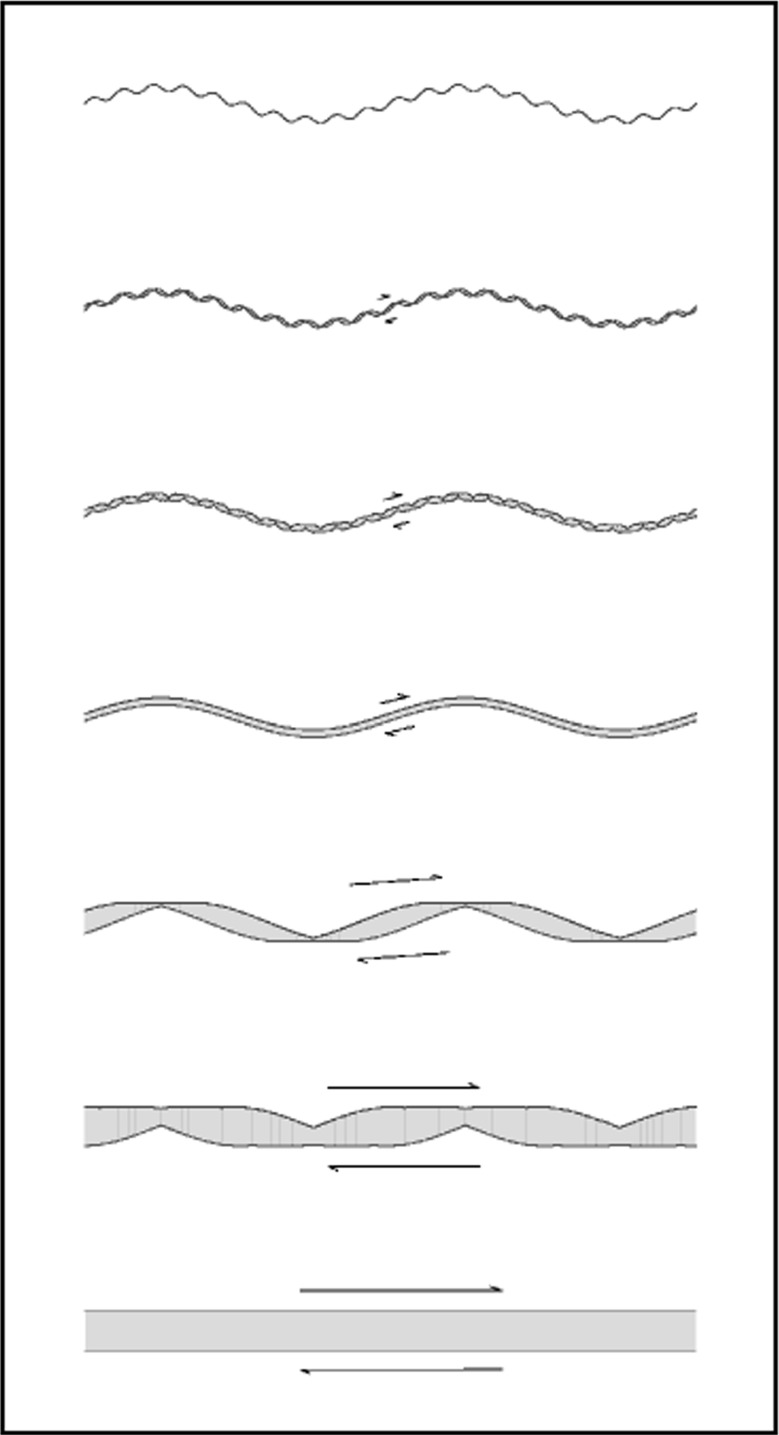
Schematic evolution of fault surface waviness and associated damage with increasing slip. A simple model with only two different wavelengths is shown

We consider another simplified model where we assume that slip *u* results in the irreversible anelastic abrasion of asperities in the scale *λ* ≈ *u*, through brittle, ductile, or plastic distributed deformation processes in the vicinity of the fault. An ideal sketch can be found in Fig. 10. In our ideal model, shear deformation during abrasion of asperities at scale *λ* takes place within a band of thickens approximately *h*(*λ*), the elevation of fault topography at a given wavelength. As slip progresses, wavelengths involved in the abrasion process become larger and larger, and the thickness of the deformation band increases. In Fig. 10, two different wavelengths are shown, together with the increasing area of shear. The deformation within the shear band is of the order of *ε*_*p*_ ≈ *h*/*u*; If shear occurs at an average plastic yield stress *σ*_*y*_, over a thickness *h*, we will have w(*u*) = *σ*_*y*_ (*h* ^2^/*u*) per unit fault area. Using *h* = *λ* ^*H*^ = *u* ^*H*^, we obtain w≈*u* ^2*H*−1^. In this case, too, the exponent of the dissipated work is quite smaller (≈0.6) than the exponent (≈1.28 obtained fog *G* ^′^ from seismological estimates. Furthermore, we note that since the strain *ε*_*p*_ ≈ *h*/*λ* ≈ *λ* ^*H*−1^ decreases with *λ* for *H* < 1, a limit where infinitesimal strain can be accommodated elastically will be reached as the slip increases, thus inducing a saturation of dissipated energy. Assuming a limit of elastic strain at 1 %, and *h* = 0.05 *λ* ^*H*^, dissipation would saturate at slip *u* ≈ 3.6 m. Such an amount of slip lies within the large end-members of the seismological catalog of Fig. 5; therefore, the lack of larger events would hinder the observation of any change in behavior.

### Fault segmentation

According to the above discussion, fault roughness described by *H* ≈ 0.8 would yield off-fault dissipation scaling with a much lower exponent (≈0.6 versus ≈1.3) than that captured by the seismological estimates of *G* ^′^ .

However, power-law roughness of faults is measured on individual, continuous segments (Bistacchi et al. 2011; Candela et al. 2012). Further complexity is induced by fault discontinuities, i.e., jogs or fault segmentation. In particular for large earthquakes, seismic ruptures have been known to span more than one fault segments, for example, in the well-documented Landers earthquake (Aochi et al. 2003, and references therein).

A fault jog is associated with the termination of an individual fault segment, and the transition of slip onto another fault segment within an echelon-type structure. As a consequence, stress concentration in the vicinity of jogs is similar to that at the tips of an isolated rupture (albeit generally larger owing to constructive interference of both segments), inducing substantial damage and dissipation.

Indeed, documented km-scale contractional jogs show an increase of up to five times in the thickness of the damage zone (Bistacchi et al. 2010, reported on the Sprechenstein fault system, Eastern Italian Alps). This additional source of dissipation and its scaling are not quantitatively explored here, but may constitute an interesting case for further modelling and for a systematic census of jog-related anelastic strain in faults.

## Conclusion

In this study, we report detailed evolution of shear stress in high slip velocity experiments on calcite-rich and silica-rich rocks which are representative end-members of the Earth crust and some, but not all, earthquake fault environments.

We show that, under the experimental conditions explored here (slip rate *V* = 3–6.5 m/s, slip acceleration $\dot {V}=6.5\text { m~s}^{-2}$ m/s^2^, normal stress 10–30 MPa): (1) frictional weakening with slip is compatible with a power law of the type *τ* ∝ *u* ^−*α*^ with *α* ≈ 0.57. (2) Weakening is typically triggered at slip velocities of *V*_*w*_ ≈ 0.4 m/s and *V*_*w*_ ≈ 0.1 m/s at normal stresses of 10 and 30 MPa, respectively; extrapolating this trend *V*_*w*_ will decrease even further at higher normal stresses. (3) Constant acceleration results in the relation between slip *u* and slip rate $V=\sqrt {2\ u\ \dot {V}}$ , with the consequence that the weakening typically starts after slip of 0.004 m<*u*_*w*_ < 0.04 m given the above *V*_*w*_ and $\dot {V}$. (4) Weakening is complete after slip of the order of *u* ≈ 1 m for silica-rich rocks, as soon as the acceleration phase ends, but up to *u* ≈ 10–15 m for calcite, well into the interval of constant slip velocity. The difference is ascribed to the frictional melting and fast sample shortening in the case of silicate-rich rocks, resulting in a rapid achievement of steady state.

We compute the equivalent fracture energy *G*_*f*_ resulting from the frictional weakening and compare it with estimates *G* ^′^ obtained for earthquakes from seismology estimates. Both are in the form of a power law of slip; however, the exponents are substantially different (*τ* ∝ *u* ^0.5^ for *G*_*f*_ versus *τ* ∝ *u* ^1.28^ for *G* ^′^). Though *G* ^′^ and *G*_*f*_ values are compatible for earthquakes with slip in the range of centimeter to meter of slip (approximate magnitudes 3<*M*_*w*_ < 7 and 10^4^ < *G* < 10^6^ J.m^−2^), they do diverge considerably for larger earthquakes, owing to the different exponent.

To explain the large exponent from seismological estimates, we attempt a preliminary analysis of off-fault dissipation due to fault non-planarity. The latter yields a scaling of dissipation with slip with an exponent between 0.6 and 0.8, which is much smaller that the seismological estimates (1.28). On the other hand, we may assume that the main energy sink is due to plastic strain associated to the stress concentration at the tip of a propagating rupture. In that case, it has been shown (Andrews 2005) that dissipation (per unit fault area) increases proportionally to fault length. According to the elementary seismological scaling relations, slip is proportional to fault length, in this case the dissipation yields a scaling exponent of 1, which is close to and possibly compatible with the seismological estimate of 1.28, given its reduced precision.

The dissipation related to rupture tips can be multiplied and amplified by the presence of fault jogs along the seismic rupture, which act as multiple stress concentrators within the main rupture area.

The striking conclusion is that, because faults are rapidly and efficiently lubricated upon fast slip initiation, the dominant dissipation mechanism in large earthquakes may not be friction but be the plastic, off-fault strain due to fault segmentation and stress concentrations in a growing region around the fracture tip.

## Appendix: Computation of *G*_*f*_

The experimental value of *G*_*f*_ is obtained by discretizing Eq. 2. Using the recorded data points, which are sampled at a time interval *dt* = 40 10^−6^ s, and using *n*, *m* to represent the time indexes, then at time *t* = *n* *d* *t* we have:

$$G_{f}(n)=\sum\limits_{m=1}^{n}\left( \tau(m)-\tau(n)\right)\left( u(m)-u(m-1)\right). $$

Note that in case of slip-hardening (transient increase of *τ*), instead of subtracting *τ*(*n*) in the above equation, we subtract the minimum observed *τ* value within the interval [1, *n*].

### Elastic stress and strain around wavy fault

The elastic stress and strain distribution for slip on an interface described by *y* = *h* sin(2*π* *x*/*λ*)(assuming that *u* ≪ *λ* and *h* ≪ *λ*) is of the form:

21 $$\begin{array}{@{}rcl@{}}\sigma_{\text{ij}}(x,y) & = & E\left( a_{ij}+b_{ij}\frac{2\pi y}{\lambda}\right)\frac{h\ u}{\lambda^{2}}\ g(2\pi x/\lambda)\\ &&\times \exp(-2\pi|y|/\lambda)+\sigma_{ij}^{0}\\ \varepsilon_{\text{ij}}(x,y) & = & \left( c_{ij}+d_{ij}\frac{2\pi y}{\lambda}\right)\frac{h\ u}{\lambda^{2}}\ g(2\pi x/\lambda)\\ &&\times \exp(-2\pi|y|/\lambda)+\varepsilon_{ij}^{0} \end{array} $$

where g is a function of periodicity 2*π* (e.g., sine or cosine), *E* is Young modulus, and u is slip. (The zero superscripts indicate background initial values of stress and strain). After Saucier, *g* = cos(2*π* *x*/*λ*) for diagonal elements of the stress and strain tensors, and sin(2*π* *x*/*λ*) for off-diagonal terms. (*a*_*i**j*_, *b*_*i**j*_) take the values (1,1), (1, −1), (0,1) for *i* *j* = *x* *x*, *y* *y*,and *xy*, respectively, and (*c*_*i**j*_, *d*_*i**j*_) take the values take the values (1−*ν*,1 + *ν*), (1−*ν*,−1−*ν*), (0,1 + *ν*) for *i* *j* = *x* *x*, *y* *y*, and *xy*, respectively. These general properties apply either to the study of lubrified interfaces in Hertzian contact with partial opening (Dundurs, Block), to the model proposed by Saucier for offset on lubrified sinusoidal interface (Saucier) or to the model including friction at the interface proposed by Chester and Chester (2000).

If we release the assumption that *u* ≪ *λ*, when slip *u* is exactly equal to one wavelength, the two half-spaces on each side of the fault are exactly in phase again, so that strain and stress perturbation cancel. As a consequence, a periodic topography will sustain a cyclic stress fluctuation which can be obtained by replacing $u/\lambda \rightarrow \frac {1}{2\pi }\sin (2\pi u/\lambda )$, and we obtain:

22 $$\begin{array}{@{}rcl@{}} \sigma_{\text{ij}}(x,y) & = & E\left( a_{ij}+b_{ij}\frac{2\pi y}{\lambda}\right)\frac{h}{2\pi\lambda}\ \sin(2\pi u/\lambda)\\ &&\times g(2\pi x/\lambda)\exp(-2\pi|y|/\lambda)+\sigma_{ij}^{0}\\ \varepsilon_{\text{ij}}(x,y) & = & \left( c_{ij}+d_{ij}\frac{2\pi y}{\lambda}\right)\frac{h}{2\pi\lambda}\ \sin(2\pi u/\lambda)\\ &&\times g(2\pi x/\lambda)\exp(-2\pi|y|/\lambda)+\varepsilon_{ij}^{0} \end{array} $$

(in the limit *u* ≪ *λ* we have $u/\lambda \approx \frac {1}{2\pi }\sin (2\pi u/\lambda )$ so that we retrieve Eq. 21. Work density *d*w induced by elementary strain *d* *ε* is *d*w = *σ*_*i**j*_ *d* *ε*_*i**j*_, with implicit summation over repeated indexes. In terms of slip, we can write $d\mathrm {w}=\sigma _{ij}(u)\ \varepsilon ^{\prime }_{ij}(u)\ du$, where the prime denotes the first derivative with respect to *u*. Here, *σ*_*i**j*_ refers to the stress variation with respect to initial condition.

Integrating the absolute value of *d*w over *u* and averaging over *x*, then integrating over all *y*, we obtain the same scaling for for both expressions (21) and (22), namely, $\mathrm {w}=f_{ij}\ E\ h^{2}/\lambda +g_{ij}\ \sigma _{ij}^{0}h$ (where $f_{ij}=\frac {1}{128\ \pi ^{3}}(\frac {1}{2}a_{ij}c_{ij}+bc_{ij}+a_{ij}d_{ij}+b_{ij}d_{ij})$ and $g_{ij}=\frac {1}{2\ \pi ^{3}}(c_{ij}+d_{ij})$) for each cycle of strain, where w is the absolute value of elastic strain work performed for each cycle of strain (*u* = *λ*). After *n* = *u*/*λ* cycles, the total work will be:

23 $$ \mathrm{w}={\Sigma}_{ij}\ f_{ij}\ E\ u\ h^{2}/\lambda^{2}+{\Sigma}_{ij}g_{ij}\sigma_{ij}^{0}\ u\ h/\lambda.  $$

$$\mathrm{w}\propto u\ \left( \frac{h^{2}}{\lambda^{2}}+A\ \frac{h}{\lambda}\right) $$

Since *h* ≪ *λ* but likely $E\gg g_{ij}\ \sigma _{ij}^{0}$, we do neglect neither term in Eq. 23, and define the dimensionless ratio $A=(E\ {\Sigma }_{ij}g_{ij}\sigma _{ij}^{0})/(\sigma _{ij}^{0}{\Sigma }_{ij}\ f_{ij})$.
